# ERRATUM

**DOI:** 10.1002/clc.23265

**Published:** 2019-09-09

**Retraction: Richard M. Fleming, Matthew R. Fleming, Gordon M. Harrington, Keith‐Thomas Ayoob, David W. Grotto, and Andrew McKusick, Long‐term health effects of the three major diets under self‐management with advice, yields high adherence and equal weight loss, but very different long‐term cardiovascular health effects as measured by myocardial perfusion imaging and specific markers of inflammatory coronary artery disease. Clin. Cardiol. Accepted Article** http://doi.org/10.1002/clc.23047.

*Clinical Cardiology*. 2018;41:1620.

DOI: 10.1002/clc.23047

In the original Retraction statement published for this article, the wording was ambiguous and did not reflect current policy of the publisher. The act of “retraction” for an article published in the unformatted, “Accepted Article” (non‐archival) format is now referred to as a “withdrawal” and therefore the wording should have been as follows:

The above article from Clinical Cardiology, posted online on September 27, 2018 in Wiley Online Library (http://onlinelibrary.wiley.com), has been withdrawn by agreement between the journal Editor in Chief, A. John Camm, and Wiley Periodicals, Inc. The article has been withdrawn due to concerns with data integrity and an undisclosed conflict of interest by the lead author.

The publisher regrets the error.

